# Multivariate classification of echellograms: a new perspective in Laser-Induced Breakdown Spectroscopy analysis

**DOI:** 10.1038/s41598-017-03426-0

**Published:** 2017-06-09

**Authors:** Pavel Pořízka, Jakub Klus, Jan Mašek, Martin Rajnoha, David Prochazka, Pavlína Modlitbová, Jan Novotný, Radim Burget, Karel Novotný, Jozef Kaiser

**Affiliations:** 1 Central European Institute of Technology, Brno University of Technology, Purkyňova 123, Brno, 612 00 Czech Republic; 2 AtomTrace a.s., Kolejní 9, 612 00 Brno, Czech Republic; 3 Faculty of Mechanical Engineering, Brno University of Technology, Technická 2896/2, 616 69 Brno, Czech Republic; 4 Sensor, Information and Communication Systems, Brno University of Technology, Technická 12, 616 00 Brno, Czech Republic; 5 Faculty of Electrical Engineering and Communication, Brno University of Technology, Technická 12, 616 00 Brno, Czech Republic; 6 BurgSys, a.s., Hněvkovského 30/65, 617 00 Brno, Czech Republic

## Abstract

In this work, we proposed a new data acquisition approach that significantly improves the repetition rates of Laser-Induced Breakdown Spectroscopy (LIBS) experiments, where high-end echelle spectrometers and intensified detectors are commonly used. The moderate repetition rates of recent LIBS systems are caused by the utilization of intensified detectors and their slow full frame (*i.e*. echellogram) readout speeds with consequent necessity for echellogram-to-1D spectrum conversion (intensity *vs*. wavelength). Therefore, we investigated a new methodology where only the most effective pixels of the echellogram were selected and directly used in the LIBS experiments. Such data processing resulted in significant variable down-selection (more than four orders of magnitude). Samples of 50 sedimentary ores samples (distributed in 13 ore types) were analyzed by LIBS system and then classified by linear and non-linear Multivariate Data Analysis algorithms. The utilization of selected pixels from an echellogram yielded increased classification accuracy compared to the utilization of common 1D spectra.

## Introduction

Laser-Induced Breakdown Spectroscopy (LIBS)^1–4^ is gaining a position among other analytical techniques based on its advantages such as instrumental simplicity, robustness, and the capability of real-time analysis of a broad range of elements, even in *in situ* and stand-off modes. LIBS has already been utilized in various applications for qualitative and quantitative elemental analysis, as has been reflected in a number of review articles^5–13^. However, along with its main drawback - the matrix effect^14^ - as a laser-ablation-based analytical technique, the performance of LIBS analysis is strongly dependent on the sensitivity of the detection system used. In general, intensified detectors are essential for temporally resolved LIBS experiments to gate-out the unwanted continuum and Bremsstrahlung radiation at the beginning of LIP formation^1^. Consequently, temporally resolved detection enables improved signal to background/noise ratios (SBR and SNR, respectively) and in turn better limits of detection (LODs). Moreover, especially in the case of multivariate classification, it is beneficial to utilize broadband echelle spectrometers to obtain more diverse spectroscopic information (numerous spectral lines from various elements).

A recent trend in the development of state-of-the-art instrumentation is in the improvement of the repetition rate, to enable high-rate shot-to-shot analysis. The repetition rate is of great interest, notably in mapping large sample surface areas. Certain LIBS devices can provide laser sampling with more than a 100 Hz repetition rate^2^, even at the level above 10 kHz, because of the utilization of high-rate DPSS or fiber laser sources^15^ but lack the possibility of temporally resolved detection. To briefly sum the information regarding the repetition rate, conventional intensified detectors are the bottleneck of overall LIBS systems. The most frequently used CCDs are capable of read out at only units or tens of full frames per second (fps), in the case of a >1Mpx chip. In that sense, CMOS detectors are significantly faster.

Due to the relatively poor historical performance of CMOS detectors, CCDs have usually been the preferred choice for high-performance scientific applications, including the intensified detectors known as ICCDs. Relatively low fps can be increased by binning, but this is suitable only for Czerny-Turner spectrometers, not for the echelle type. Achieving fps on the order of kHz is possible by repeatedly integrating the signal on a chip before readout, but saturation strongly limits this feature/possibility. Recently, available scientific CMOS (sCMOS) detectors have proven that the CMOS technology has matured and that it is catching up rapidly with CCDs in many parameters while retaining the benefit of a high fps rate. LIBS could thus profit from the high fps and additionally from the possibility of direct readout of single pixels. This implies that the sCMOS detectors could equalize the repetition rate of high-rate laser sources. This essential feature directly arises from the sCMOS technology itself, when detection is limited to sub-windows (so called regions of interest) and readout may reach hundreds of fps^16, 17^.

This manuscript proposes and advocates a methodology enabling high-repetition-rate LIBS analysis. For that purpose, it is necessary to select areas of interest in obtained echellograms. Each Laser-Induced Plasma (LIP) spectrum is unique in its composition and characterizes the interaction spot on the sample surface. Thus, a LIP spectrum is considered to be the so-called chemical fingerprint representing the investigated sample. In general, it is convenient to cover a broad range of wavelengths, *i.e*., to have a wide variety of characteristic spectral information stored in a LIP spectrum^18^. Considering a high-repetition-rate LIBS system and a large number of spectral variables per detected spectrum, such analysis results in bulky data sets. Thus, it is beneficial to use Multivariate Data Analysis (MVDA) algorithms (linear or non-linear) to process the multidimensional data, *i.e*., data mining^19, 20^. The issue of multivariate classification of LIP spectra has recently gained particular attention in the LIBS community^4, 5, 7, 11, 12, 20^.

There are several ways to treat raw spectra prior to MVDA analysis^20^. In the first step, it is usually convenient to mitigate the fluctuation in the data, *i.e*., exploiting outlier-filtering algorithms^21^ or normalization strategies^22–24^. One should also decide whether to use the whole spectral range, the selected parts of interest, or only the intensity of several (matrix and minor) lines^25^. The last of those alternatives significantly lowers the number of variables, from thousands to tens. The reasoning for using the whole spectral range is in the potential importance of minor and trace elements and their lines or even the background^26^. On the other hand, exploiting only a limited number of spectral lines mitigates the impact of noise and in turn improves the classification accuracy.

In our work, we focused on the utilization of echelle spectrometers because they provide a broad range of wavelengths (spectral information), which is beneficial mainly, but not only, for classification purposes. In common practice, an obtained echellogram (detected 2D image) is converted to a common 1D spectrum (intensity *vs*. wavelength). The converting algorithm (transforming the 2D echelle image to the 1D spectrum; provided by the spectrograph manufacturer) gives the best trade-off between the relative efficiency of the grating and its respective spectral resolution. It is noteworthy that the conversion of an echellogram to a 1D spectrum is a standard approach in LIBS but can lead to a possible loss of information and thus a degradation of the model performance.

To date, direct utilization of echellograms in the LIBS community remains rather unusual. Larsson *et al*.^27^ compared the performance of a Partial Least Squares Discriminant Analysis (PLS-DA) algorithm where either 1D spectra or raw echellograms were fed to the algorithm as the input variables. Their aim was to show the impact of variable reduction on the discrimination capability. First, the classification performance of the model showed better results for normalized 1D spectra over the selection of the highest peaks from such spectra. Second, cropping and binning of 2D image pixels was utilized. Better results were observed when the PLS-DA algorithm was applied to the half image with hard binning (32 × 32 pixels) than to the 1D spectra. This result showed that strong data reduction (retaining only the effective part of the echellogram) is acceptable to maintain the same classification accuracy. Moreover, it demonstrated that there is a significant loss of information during the echellogram-to-1D spectrum conversion, which is more time consuming than simply saving the full resolution (uncropped and unbinned) echellogram image.

The present work is aimed at the direct utilization of raw echellograms for classification purposes. The experiment was designed to demonstrate the improvement in repetition rate using conventional LIBS instruments, namely, intensified detectors. Thus, we propose a new approach in which only certain sub-windows of the full frame image are selected for readout (*i.e*., pixels of interest in the echellogram, spectral variables). Moreover, those sub-windows can be spatially separated. This is another advancement that is enabled only by the sCMOS detector. Afterwards, the echellogram is read out only for the selected pixels, and truncated information may be stored with a higher repetition rate. The performance of the proposed approach is compared with the utilization of classical LIBS 1D spectra (intensity *vs*. wavelength) for classification of LIBS data (sedimentary ores). Simple linear and advanced non-linear MVDA algorithms are used for the classification purposes.

## Results

**Figure 1:**
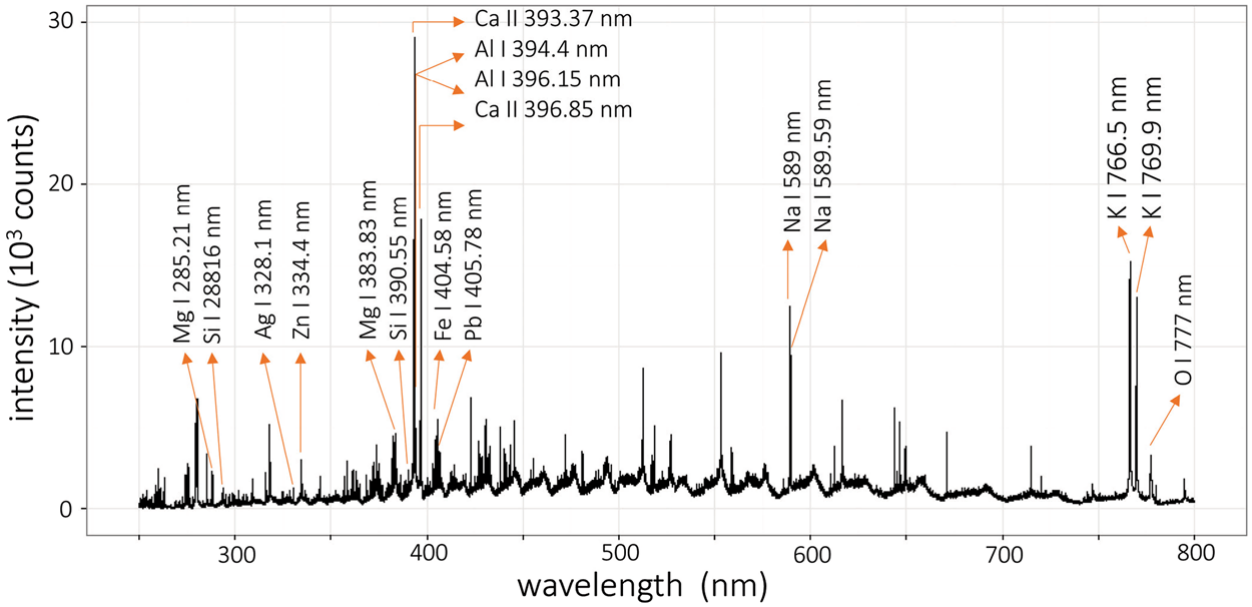
LIP spectrum of the O131b sample.

**Figure 2:**
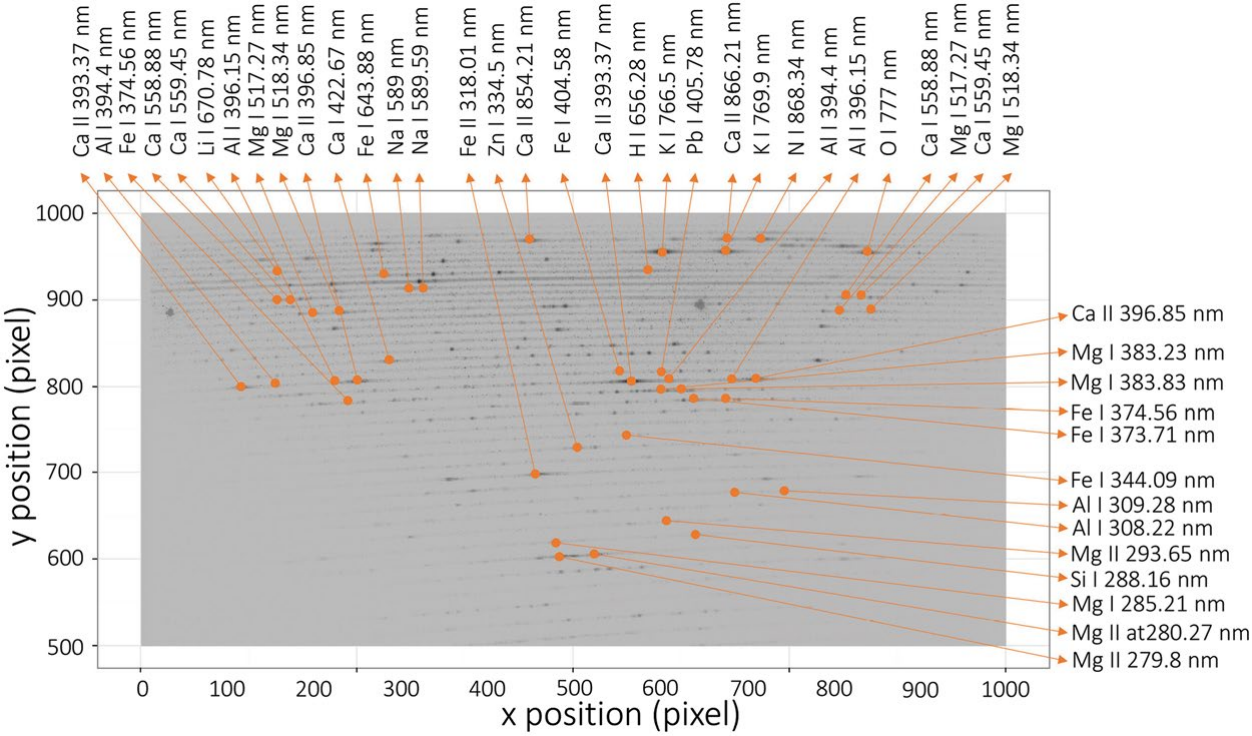
Echellogram of the O131b sample.

Prior to the MVDA clustering of samples, the 1D spectra and echellograms were inspected in detail as is common in a standard LIBS experiment. We believe that plotting the characteristic spectra of each selected sample matrix is redundant. Therefore, they are not given in the text except for the spectrum of sample O131b (Zn-Pb-Ag sulfide ore). This sample was selected because it contains a certified abundance of Ag, Al, Ca, Fe, Mg, Pb, and Si in wt.%. Both the line spectrum and the echellogram, depicted in Figs 1 and 2, were obtained as the mean of all detected spectra or echellograms, respectively, during the measurement of this sample. No normalization was employed for the data visualization. Certain lines of the aforementioned major and minor elements are highlighted in both figures. The use of an echellogram is rather unconventional for the analysis of LIBS data, and therefore, more lines were assigned. It is worth mentioning that several wavelengths can be observed at two distinct places in the echellogram, for instance, lines of Ca II 393.37 nm, Al I 394.4 nm, Al I 396.15 nm, Ca II 396.85 nm, and others. This is an example of the way in which the echelle spectrometers scatter the light (refer to the specialized literature for a more detailed description^28^). Afterwards, the echellograms were normalized using histogram equalization. In this pre-processing step, the data were rescaled into a more balanced dynamic range.

In the next step, 225 pixels from the echellograms were selected using the proposed approach. The number of selected pixels was then further reduced to 86 or even only 21 pixels. It is worth mentioning that those pixels (the most informative features in the echellograms) were not neighboring and were scattered across the image. Their detection is thus enabled only through using the sCMOS technology, as a conventional CCD offers only cropping of image areas. This pre-processing step significantly lowers the number of variables per data set, by four orders of magnitude. Naturally, this variable reduction leads to a significant improvement in detector readout time and could equalize the repetition rate of the high-rate laser sources (>1 kHz)^16, 17^. This is, of course, based on the assumption that the readout is done only from certain sub-windows of the full frame image.

**Figure 3:**
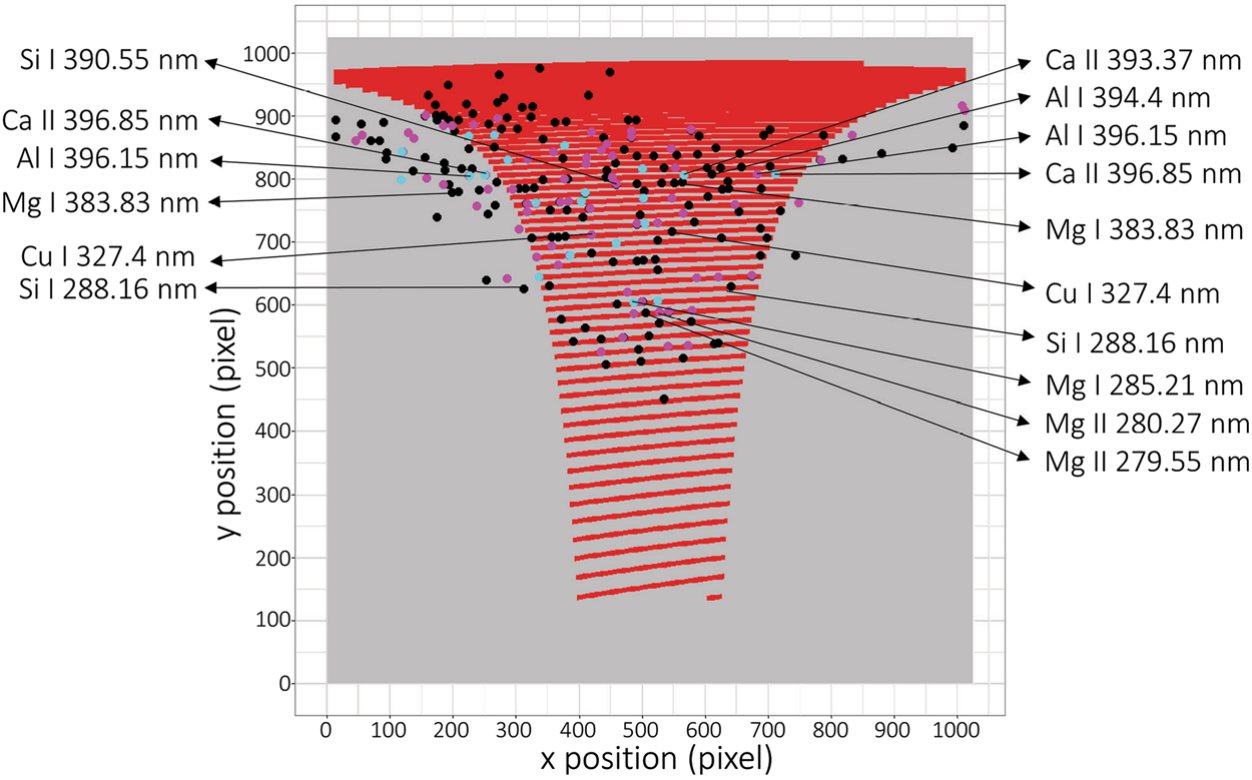
Diffraction orders of the echelle spectrometer used for the readout of a typical LIBS spectrum (red lines) and positions selected based on the proposed approach (225 pixels - black dots, 86 pixels – pink dots, 21 pixels – cyan dots).

Selected valuable pixels are highlighted in Fig. 3 together with diffraction orders (red colored lines), *i.e*., the position in the echellogram from which the spectroscopic information is read out. Apparently, those orders do not cover the whole range of the echellogram (1024 × 1024 pixels), making a considerable number of pixels useless, namely, the bottom part of the echellogram, where the orders refer to wavelengths below 250 nm. Below this range, the spectrometer offers a good resolution but poor spectral efficiency (together with the detector). Thus, the bottom half of the echellogram can be, in our case, instantly truncated for its redundancy. This in turn improves the readout speed of the detector.

In Fig. 3, spectral lines are assigned to several points selected by the proposed approach. The assigned elemental lines show the largest variation in the data set, and thus, they tend to be the most valuable. The selected points are scattered mainly in the upper part of the echellogram, proving again the redundancy of the bottom part. Despite the optimized algorithm for transferring the echellogram into 1D spectra, as described above, the highlighted orders do not cover all the points selected in the pre-processing step. This issue suggests an inefficient transfer of information into the 1D spectrum and a possible loss of analytical performance.

**Figure 4:**
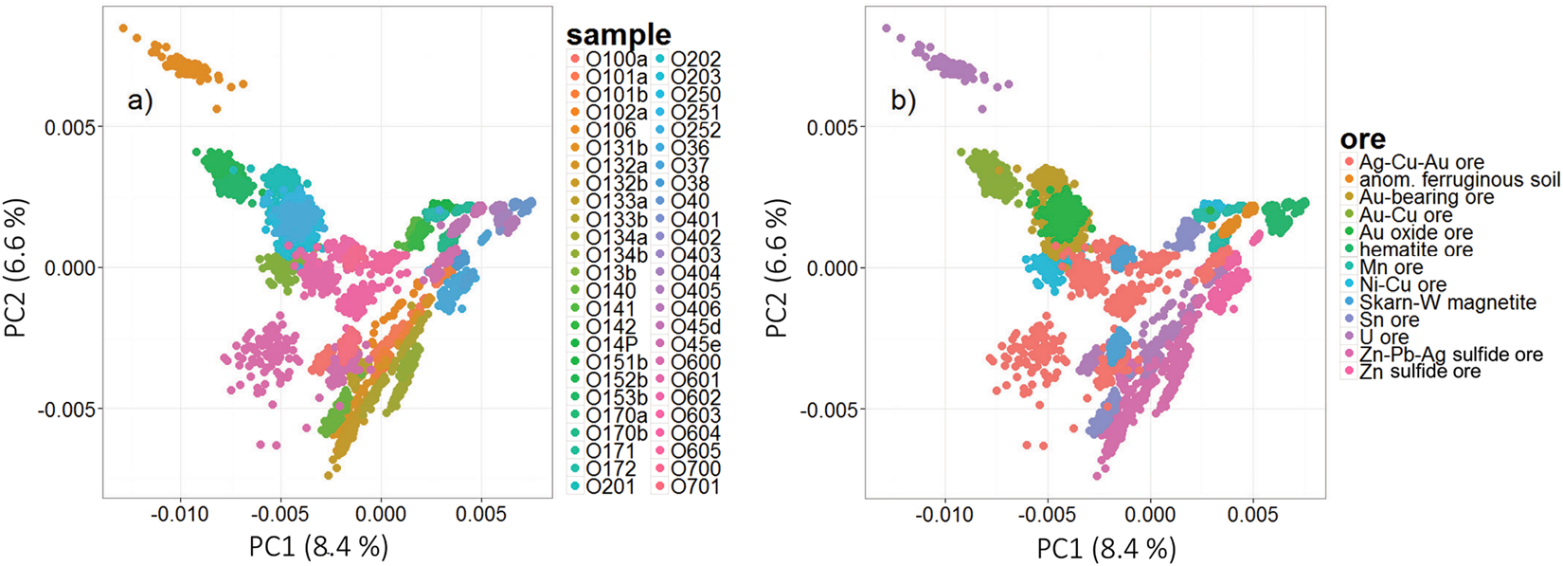
PCA scores for the analysis yielded from 1D LIBS data of sedimentary ore CRMs, where data were classified according to (**a**) individual sample and (**b**) specific ore.

In the LIBS literature concerning MVDA classification, a data set is first visualized prior to any further analysis to show any potential scattering of the detected data into clusters. For that purpose, PCA was applied to the whole data matrix **X** constructed from the detected 1D spectra of all 50 samples. The data were row-wise normalized to total intensity as stated above and then column-wise mean-centered, as suggested in our recent publication^24^. The resulting scores of the first two principal components are depicted in Fig. 4. The data set was distributed into a) 50 classes according to the individual samples and b) 13 classes according to the respective ore type of each sample. Despite the difficult-to-read legend, individual samples form compact clusters that are well distinguished in the PC space. This is also confirmed by the obtained classification accuracy. The discrimination of samples is assigned to their unique chemical composition (inter-class and intra-class). Each sample is represented by several outlying points, but those were not filtered in order to stay close to real life application. It is also noteworthy that the ore types are not compact and are not fully separated from each other. This is due to the compositional variation of the individual samples. For instance, sample O106 is distinctly separated from the rest of the U ore samples. Thus, the divisions between individual ore types must be accepted carefully. It must be stressed that only the first two principal components are printed. However, for complete understanding of the distribution of data in multivariate space, more PCs have to be investigated.

**Figure 5:**
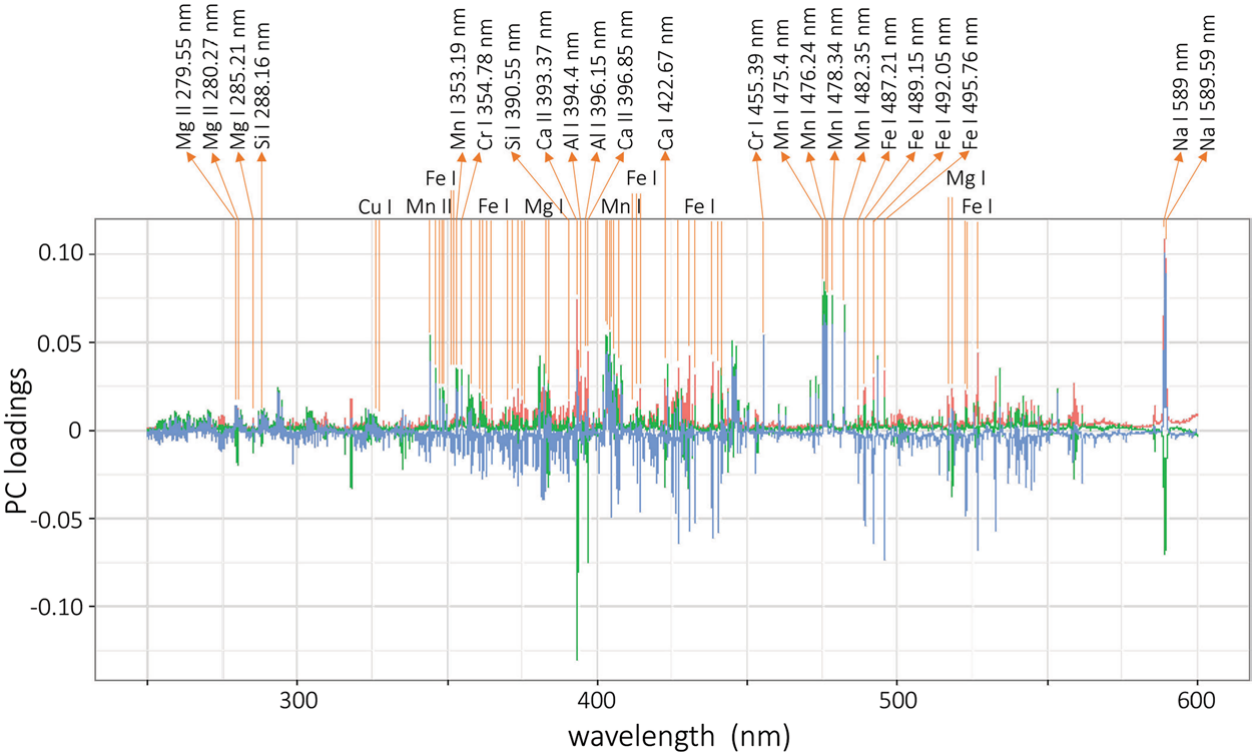
PCA analysis yielded from sedimentary ore CRM LIBS data: loadings, where PC1 is red, PC2 is green and PC3 is blue.

PC loadings provide information about the importance of individual variables responsible for sample set clustering. The loadings of the first three principal components, describing 20% of the total variance within the data set, are depicted in Fig. 5. Only wavelengths ranging from 250 to 600 nm are depicted for better readability of the plot. Below 250 nm, the sensitivity of the utilized detection system rapidly decreases. Over 600 nm, the number of important lines is significantly lower than in the depicted range. The highest loading values in the depicted range are possessed by the Na, Ca, and Al lines, followed by the Fe, Mn, Cr, and Mg lines. The oxygen line at 777 nm and the potassium lines at 766.5 and 769.9 are also of considerable interest (not shown in the figure), while having higher loading values. The observed analytical lines are natural lines of the matrix and minor elements possessing the greatest variation among the samples’ contents.

After the visualization of the 1D spectra, we intended to visualize the data set composed of the whole echellograms using PCA. For those purposes, each obtained echellogram image (matrix of 1024 × 1024 pixels/intensity information) was melted into a single vector of 1 048 576 length. Such vectors were then organized into a data matrix, where rows (5000 in total) were assigned to individual melted echellogram images/measurements and columns were pixels/variables. However, a matrix of those dimensions could not be processed in the R software, installed on an office PC with 32 GB of RAM. To reduce the dimensionality, the echellograms were cropped, and their bottom parts were sacrificed for the sake of computation. However, even after doing so, it was not possible to estimate PCA scores and loadings for the truncated data matrix even. Therefore, PCA analysis of the echellogram images, without variable down-selection, is missing.

**Figure 6:**
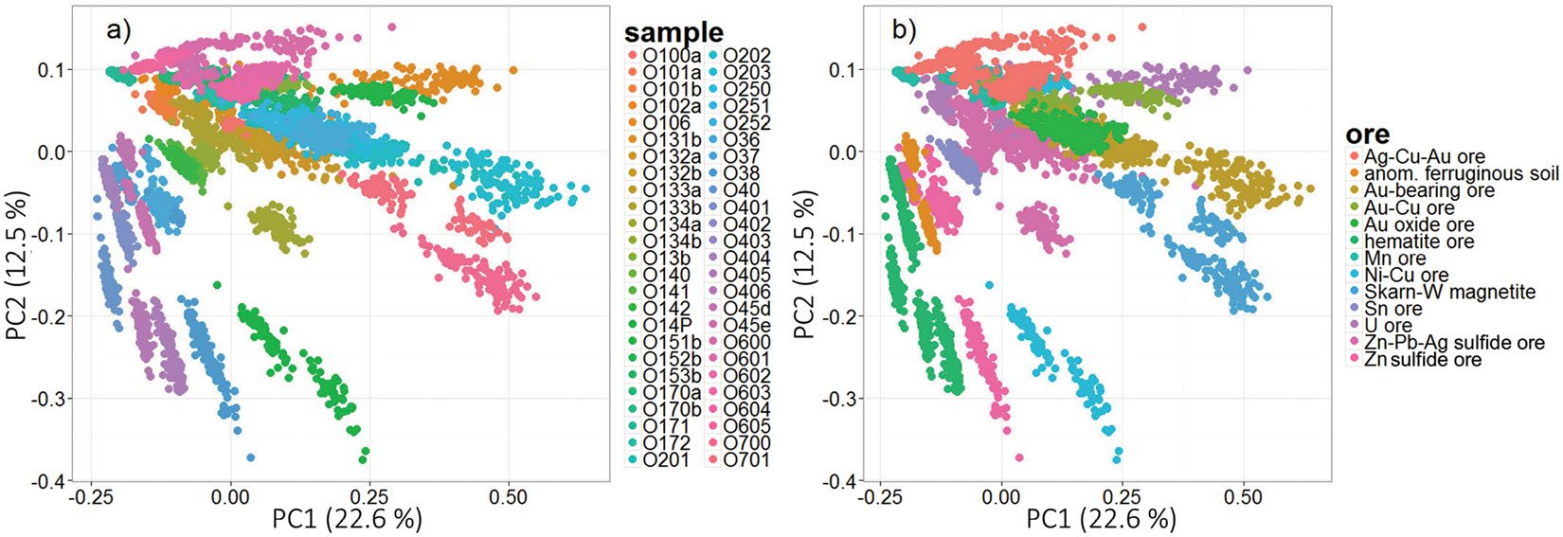
PCA scores of 225 selected points from echellograms, where data were classified according to (**a**) individual sample and (**b**) specific ore.

The selected 225 data points/pixels from the echellograms significantly reduced the dimensions of the data matrix. Afterwards, the computation was naturally faster and less extensive. The first two PCA scores estimated from the data matrix are depicted in Fig. 6. The PC space was depicted for data distributed according to a) samples and b) ore types. When the distribution according to the ore type is considered for more descriptive investigation, the data are scattered in the PC space in more compact and distinctly separated clusters. This is in strict contradiction to the results observed when the 1D spectra were under investigation in PC space, Fig. 4. This suggests that selecting the most informative pixels is beneficial for data processing. The first three principal components describe ~46% of the total variance. This is much more than in the previous case where 1D spectra were coupled to PCA. The higher variance is obtained because of the lower number of variables and the absence of noise. PCA analyses of the data sets constructed from 86 or 21 pixels are not provided in this manuscript.

In the last step of our analysis, the obtained LIBS data, 1D spectra and the data points selected from echellograms using the proposed approach were classified utilizing linear (L2R LR) and non-linear (SVM) MVDA algorithms. The classification accuracy for each case was proposed as a figure of merit and was estimated from confusion matrices. The obtained results are summarized in Table 1. The non-linear algorithms yielded slightly higher accuracy, which was expected and is also in agreement with literature^20^. This table was constructed for the results obtained for data distributed to classes according to sample (50) and respective ore type (13). The overall trend shows that classifying the data according to ore type yields higher accuracies, regardless of the MVDA algorithm used. This relates to the fact that data from the samples of the same ore type slightly overlap, which may cause misclassification. Most falsely classified data were recorded with classes that are relatively close to each other: in the case of 50 classes, several data points of class O132a were wrongly predicted to be O132b, and contrariwise, O132b was wrongly predicted as O132a. Also, classes O402 and O403 had mutually wrong predictions. In the case of 13 classes, mutual wrong predictions are present, namely, between the Au oxide ore and Au-bearing ore classes.

**Table 1:** Classification results of sedimentary ores using linear (L2R LR) and non-linear (SVM) MVDA algorithms. Prior to classification, the data set was distributed into 50 classes according to individual samples and then according to respective ore type. The selection of pixels was provided from the echellograms as described in detail in the text. The best results yielded during the classification process are in bold.

Data	MVDA	Dimensionality/variables	Accuracy/%	Classes
1D spectra	SVM	35000 × 5000	96.5	50 samples
Selection of 225 pixels	SVM	225 × 5000	**98.13**	50 samples
Selection of 86 pixels	SVM	86 × 5000	97.7	50 samples
Selection of 21 pixels	SVM	21 × 5000	96.5	50 samples
1D spectra	L2R LR	35000 × 5000	95.5	50 samples
Selection of 225 pixels	L2R LR	225 × 5000	97.2	50 samples
Selection of 86 pixels	L2R LR	86 × 5000	97.3	50 samples
Selection of 21 pixels	L2R LR	21 × 5000	92.5	50 samples
1D spectra	SVM	35000 × 5000	98.9	13 ore types
Selection of 225 pixels	SVM	225 × 5000	**99.2**	13 ore types
Selection of 86 pixels	SVM	86 × 5000	99.13	13 ore types
Selection of 21 pixels	SVM	21 × 5000	98.5	13 ore types
1D spectra	L2R LR	35000 × 5000	98.9	13 ore types
Selection of 225 pixels	L2R LR	225 × 5000	99.3	13 ore types
Selection of 86 pixels	L2R LR	86 × 5000	98.9	13 ore types
Selection of 21 pixels	L2R LR	21 × 5000	95.4	13 ore types

In general, non-linear algorithms are capable of modeling LIBS data more accurately. This is usually explained as follows. The relations between intensity and elemental content do not have to be strictly linear in typical LIBS data, since the spectral line intensities are affected by the matrix effect and saturation. On the other hand, a lower number of variables (for the case of selected pixels) results in higher accuracy of the MVDA classification, due to omitting noise from the computation. However, further lowering the numbers of selected pixels (from 225 to 86 and even to 21) leads to lower accuracies. This is natural for samples composed of a higher number of different elements. It is worth mentioning that using only 21 pixels still yields excellent classification.

Clearly, there is a certain balance between classification accuracy and the number of spectral variables (either pixels in the echellogram image or spectral lines in the 1D spectrum). A high number of variables could also arise from noise, confusing the classification. However, the classification accuracy can also be low due to an insufficient number of variables, *i.e*., insufficient information carried by the data. It is also noteworthy that classification accuracy could yield excellent results provided that the number of variables of the 1D spectra was reduced (*i.e*., noise was omitted). There are basically three obstacles: a) there is no accepted algorithm for converting to a 1D spectrum from an echellogram, b) a 1D spectrum has already undergone selection from a 2D echellogram image, and c) further variable down-selection of a 1D spectrum is an extra step prolonging the analysis (this also increases the number of steps in the methodology). However, this step is undesirable in terms of the proposed approach directly using echellogram images or their selected pixels.

## Discussion

In this work, we developed a methodology for the direct utilization of echellograms in LIBS experiments. Thus, there is no need to transform a 2D image into a 1D laser-induced plasma spectrum. This non-conventional approach avoids working with typical line spectra. Instead, information detected in individual pixels (ICCD, EMCCD or even sCMOS chips) is directly exploited in the LIBS analysis. Thus, this methodology is based on a significant reduction in the data (more than four orders of magnitude) that must be read from the total original image on the chip. In general, lowering the 2D image size (ICCD, EMCCD or sCMOS chip) directly leads to an increase in readout speed and thus to a higher repetition rate of the LIBS system. Notably, when using the sCMOS detector, the pixels of interest can be distinctly scattered across the whole chip. This is in contrast with ICCD and EMCCD detectors, where the image size can be truncated but must remain intact.

The proposed methodology was demonstrated on the analysis and classification of various sedimentary ores. Data (echellograms) were obtained using an EMCCD detector and then systematically down-scaled prior to multivariate classification. Half of the echellogram image contains wavelengths under 250 nm, where the system lacks sensitivity. Therefore, the data can be instantly truncated, consistent with the literature. Consequently, most important pixels (*i.e*., spectral lines) carrying the highest amount of variance within the data set were localized using the median image approach. The analytical information was ultimately reduced from the initial 2 megapixel image to tens of non-neighboring pixels.

Following the truncation of the echellogram, it is necessary to read out only tens to hundreds of pixels from the total image to still conserve the classification accuracy of the LIBS system. Thus, 225, 86, and even 21 points were selected from the 2D image using the proposed methodological approach. The resulting reduced data sets were coupled to linear and non-linear MVDA algorithms for classification. The yielded performances were compared to the classification accuracy of samples through using 1D spectra. Moreover, non-linear MVDA algorithms proved superior to linear MVDA algorithms regardless of the input data sets, in agreement with the literature.

The samples were measured using an EMCCD detector, and complete 2D images were stored during the low-repetition-rate LIBS experiment. The proposed methodology was designed and proved only in the data processing stage. However, it must be stressed that it is reasonable to expect a significant increase in the repetition rate (over 1 kHz) of the LIBS system when the proposed methodology is implemented together with an sCMOS detector. The validity of this assumption is, of course, based on the fundamental properties of sCMOS detectors.

The proposed methodology is general and can be extended to other classification problems. The extension of this method to specific problems will lead to the creation of so-called libraries of regions, where echellogram regions will be coupled directly to elemental information. These libraries might provide a new way of processing data in LIBS.

## Methods

### Samples

The sample set consisted of 50 certified reference sedimentary ores (distributed in 13 ore types) produced by OREAS (Australia) with significantly varying concentrations of matrix, minor and trace elements. The elemental abundances of selected samples are given in Table 2. A small amount of each sample, provided as a homogeneous powder, was pressed with 18 MPa applied force over the tablet surface using a manual hydraulic press. Samples were measured in the form of pressed pellets with a 12 mm diameter (no binder was used).

**Table 2:** Table containing the compositions of selected sedimentary ores as provided by the online catalogue of the producer, OREAS (Australia). Only selected elements are presented, and their contents are in wt.%. Samples 201, 202, 203, and 250 (all of them belonging to the Au-bearing ore) are not shown in the table, because no information about the content of the selected elements is present in the catalogue.

sample	ore	Al	Ca	Cr	Cu	Fe	K	Mg	Na	Pb	Si	Ti
100a	U ore	—	—	—	—	4.66	3.94	0.84	—	—	—	0.24
101a	U ore	—	—	—	—	11.06	2.34	1.23	—	—	—	0.40
101b	U ore	—	—	—	—	10.80	2.42	1.23	—	—	—	0.39
102a	U ore	—	—	—	—	5.78	3.64	1.36	—	—	—	0.17
106	U ore	—	—	—	—	—	1.59	—	—	—	—	—
131b	Zn-Pb-Ag sulfide ore	2.31	5.49	—	—	5.71	—	4.29	—	1.90	24.76	—
132a	Zn-Pb-Ag sulfide ore	2.07	5.24	—	—	7.80	—	3.87	—	3.66	21.48	—
132b	Zn-Pb-Ag sulfide ore	2.02	5.15	—	—	7.81	—	3.85	—	3.88	21.34	—
133a	Zn-Pb-Ag sulfide ore	1.88	4.05	—	—	7.92	—	3.09	—	4.90	19.24	—
133b	Zn-Pb-Ag sulfide ore	1.83	4.01	—	—	8.21	—	3.07	—	5.20	19.11	—
134a	Zn-Pb-Ag sulfide ore	0.69	4.56	—	—	12.32	—	2.85	—	12.95	8.29	—
134b	Zn-Pb-Ag sulfide ore	0.59	4.41	—	—	12.69	—	2.77	—	13.36	7.27	—
13b	Ni-Cu-PGE	8.41	5.57	0.87	0.23	8.41	2.30	3.01	1.67	—	22.90	0.71
140	Sn ore	—	—	0.15	—	—	—	—	—	—	—	—
141	Sn ore	—	—	—	0.25	—	—	—	—	—	—	—
142	Sn ore	—	—	—	0.15	—	—	—	—	—	—	—
14P	Ni-Cu ore	2.26	0.99	—	1.00	37.10	0.87	0.28	0.58	—	—	0.25
151b	Au-Cu ore	7.84	2.02	—	0.18	3.53	1.23	1.62	2.22	—	-	0.31
152b	Au-Cu ore	8.02	1.97	—	0.38	3.73	1.06	1.69	2.34	—	—	0.28
153b	Au-Cu ore	7.94	1.83	—	0.68	3.86	1.16	1.64	2.46	—	—	0.29
170a	Mn ore	1.18	0.06	—	—	—	—	0.18	—	—	6.35	—
170b	Mn ore	1.03	0.19	—	—	—	—	0.26	—	—	5.31	—
171	Mn ore	1.94	0.06	—	—	3.66	—	0.17	—	—	13.90	—
172	Mn ore	1.54	0.07	—	—	3.83	—	0.11	—	—	7.89	—
251	Gold oxide ore	1.31	0.87	—	—	5.05	0.11	1.73	0.19	—	—	0.17
252	Gold oxide ore	1.32	0.84	—	—	4.97	0.12	1.66	0.17	—	—	0.16
36	Zinc sulfide ore	—	—	—	—	20.68	—	—	—	0.58	—	—
37	Zinc sulfide ore	—	—	—	—	23.76	—	—	—	0.62	—	—
38	Zinc sulfide ore	—	—	—	—	21.28	—	—	—	0.59	—	—
40	Hematite ore	0.03	0.01	—	—	66.72	—	0.01	—	—	2.60	—
401	Hematite ore	0.62	0.07	—	—	45.63	—	0.05	—	—	13.93	—
402	Hematite ore	0.66	0.06	—	—	48.41	—	0.06	—	—	11.07	—
403	Hematite ore	0.70	0.08	—	—	52.30	—	0.06	—	—	7.66	—
404	Hematite ore	0.79	0.07	—	—	55.10	—	—	—	—	4.41	—
405	Hematite ore	0.60	0.14	—	—	58.00	—	—	—	—	4.69	—
406	Hematite ore	0.30	0.11	—	—	61.40	—	—	—	—	4.46	—
45d	Anomalous ferruginous soil	8.15	0.19	0.05	0.04	14.52	0.41	0.25	0.10	—	—	0.77
45e	Anomalous ferruginous soil	6.78	0.07	0.10	0.08	24.12	0.32	0.16	0.06	—	—	0.56
600	Silver copper gold ore	6.78	1.88	—	0.05	2.38	1.80	0.77	0.59	—	—	0.24
601	Silver copper gold ore	6.30	1.31	—	0.10	2.48	2.10	0.39	1.45	—	—	0.18
602	Silver copper gold ore	4.37	0.62	—	0.52	2.24	0.68	0.20	0.46	—	—	0.21
603	Silver copper gold ore	3.98	0.32	—	1.00	2.92	0.62	0.08	0.43	—	—	0.19
604	Silver copper gold ore	5.82	0.74	—	2.16	3.02	1.32	0.21	0.84	—	—	0.19
605	Silver copper gold ore	5.43	0.28	—	5.02	3.76	1.04	0.05	0.58	—	—	0.18
700	Skarn tungsten magnetite	5.57	5.55	—	0.20	15.57	1.57	1.00	1.21	—	—	0.18
701	Skarn tungsten magnetite	6.32	3.62	—	0.49	23.02	2.57	0.72	0.69	—	—	0.15

### LIBS system

**Figure 7:**
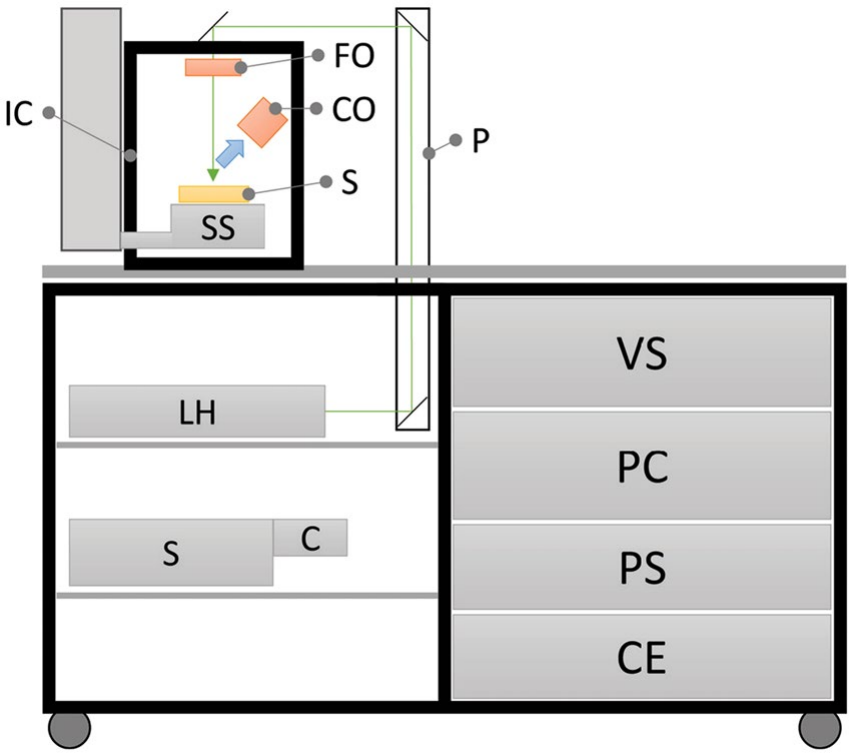
Schematic diagram of Sci-Trace (AtomTrace, CZ), where LH: laser head, S: spectrometer, C: camera/detector, VS: vacuum system (gas purge), PC: computer, PS: laser power supply, CE: control electronics, P: periscope with reflective mirrors, SS: 3-axes sample stage, S: sample, IC: LIBS interaction chamber, FO: focusing optics, and CO: collecting optics.

All LIBS measurements were performed using the Sci-Trace instrument (AtomTrace, Czech Republic), schematically depicted in Fig. 7. The Sci-Trace is a comprehensive LIBS setup, which consists of the instrumentation compartment and the LIBS Interaction Chamber^29^ mounted on the top of the optical workbench. A laser (532 nm, 10 ns, flash-lamp operated at 20 Hz; CFR Ultra, Quantel, France) was used as the ablation source; its pulses were guided *via* reflecting mirrors and a focusing lens (24.5 mm; Sill Optics, Germany) to the interaction region on the sample surface. The focused laser beam induced a luminous LIP, and its characteristic radiation was collected using a combination of 1“ UV lenses (100 mm CaF2 and 75 mm UVFS; Thorlabs, USA) leading to the entrance slit of the echelle spectrometer (190–1100 nm, 5000 *λ*/Δ*λ*; Emu-65, Catalina Scientific, USA) *via* optical fiber with a 400 *μ*m core diameter (Thorlabs, USA). Spectrally resolved radiation then illuminated the EMCCD detector (200–1000 nm, max 30 Hz repetition rate; Falcon Blue, Raptor Photonics, Ireland), and 2D images (echellograms) were downloaded. The temperature surrounding the echelle spectrometer and EMCCD detector within the instrumentation compartment was continuously controlled by the Sci-Trace electronics to mitigate the possibility of thermal shifts on the EMCCD chip. The timing of the experiment was controlled using a digital delay generator (Sapphire 9200, Quantum Composers, USA). The overall LIBS system was operated with the AtomChamber suite. The AtomAnalyzer suite (both AtomTrace, Czech Republic) was used for preliminary operations on the obtained data. The spectra were processed utilizing MVDA algorithms in BDesigner (BurgSys software, Czech Republic). R software was utilized for visualization of i) the echellograms and ii) the data sets in low-dimensional space from principal component analysis (PCA).

The sample set was analyzed under ambient conditions with air flow directed collinearly with the incident laser pulse. The continuous flow of air cleared the interaction region of the aerosol created during the laser ablation and thus prevented the creation of any pre-sparks. Each sample was measured 1000 times to provide solid statistics at 100 spots on the sample surface. Ten consecutive laser pulses were introduced to each spot, and the obtained echellograms were accumulated in the software during the data pretreatment to mitigate potential signal fluctuation. The echellograms were acquired as characteristic signals of individual LIPs. Naturally, the 1D spectra were derived from the respective echellograms by the software (Kestrel, Catalina Scientific, USA). The spacing between the interaction spots was set to 200 microns to avoid crater overlap. The exposition time of the EMCCD camera was set to 50 *μ*s (lowest possible) with a delay of 500 ns from the beginning of the laser ablation. All acquisition parameters are summarized in Table 3.

**Table 3:** Experimental settings.

Parameter	Settings
Laser energy/mJ	50
Crater diameter/*μ*m	100
Irradiance/GW · cm^−2^	~64
Gate delay/ns	500
Gate width/*μ*s	50
Spacing between spots/*μ*m	200
No. of samples	50
Spectra per sample	1000
Accumulation	10
Total no. of spectra	5000

### Conversion of echellograms

There are few works utilizing raw echellograms, despite them containing considerably more information than conventional 1D spectra. However, echellograms are not always accessible, and their utilization leads to dramatic increases in data volume. The idea of direct echellogram utilization was originally suggested by Larson *et al*.^27^ They proved the superiority of echellograms, especially after cropping and binning, over the conventional utilization of 1D spectra for classification purposes. A significant reduction in the data readout from the ICCD and transfer times was yielded by binning the pixels and/or cropping the image, which could induce a higher sampling rate.

The key concept of this work builds on a selective readout of the pixels of interest. Those pixels may not be in direct contact and therefore may be randomly scattered across the full echellogram. This approach cannot be directly achieved when utilizing an ICCD (leaving the selection to post-processing), but single-pixel readout is an essential feature of the sCMOS detector. Adding additional value to our approach, certain spectral features are detected with echelle spectrographs in multiple locations but with different spectral resolutions and relative efficiencies. Thus, the conversion of echellograms could still be improved. Despite that, we assume that the obtained 1D spectrum is a well-balanced conversion of data from the detected echellogram. This trade-off between the relative efficiency of the grating and the respective spectral resolution is performed by the software provided by the manufacturer. The software conversion of the echellogram results in the intensity *vs*. wavelength 1D spectrum, where 10^6^ data points are reduced to 4 · 10^4^ (4%). This suggests either a loss of information or noise depending on the selection of the areas from which the spectral line intensities are transferred for further computation.

To conclude, acquiring the complete echellogram and converting it into a 1D spectrum results in limited sampling frequency. This issue is targeted in the presented manuscript. The main contribution of this paper is a new algorithm that shows the superiority of direct pixel selection over the utilization of 1D spectra. In the proposed approach, only tens or hundreds of echellogram pixel values are needed from a total 1024 × 1024 = 1 048 576 pixels. The proposed methodology may lead, as discussed above, to the mitigation of the readout times based on sCMOS features, and thus, an increase in the LIBS system repetition rate is consequently expected.

### Data pre-processing for classification

First, the case of 1D spectra is discussed, as it is more typical for LIBS analysis. We used the total spectral range with no masking or spectral line selection because the 1D spectrum itself is already a well-balanced selection of lines/variables from the original echellogram. In this case, there are 35 000 spectral variables in the spectrum, ranging from 200 to 1000 nm. We are also aware that a significant amount of noise is introduced into the consequent computation. The data matrix **X** was constructed for the classification of 1D spectra, measurements/spectra were assigned to rows, and variables/wavelengths were assigned to columns. The detected intensities in each spectrum were then normalized to the total detected intensity of that particular spectrum. This normalization approach divides each value in a row by the total sum of row values. The normalized data matrix was then processed with MVDA algorithms.

The detected raw echellograms were pre-processed prior to MVDA classification. First, images were normalized by a histogram equalization procedure^30^ to normalize obtained pixel intensities and mitigate signal fluctuation. Histogram equalization is a technique for adjusting image intensities via dynamic range enhancement, which can be considered as the normalization of data values to the range of intensities 0–1. The most frequent intensity values are linearly spread to gain higher contrast with respect to areas with lower local contrast. This equalization normalizes the measured values and reduces the effect of sensor settings (*i.e*., short or long exposure time). Throughout the experiments, this process demonstrated its positive effect on the overall accuracy.

The next step was to locate the most information-rich pixels in the echellogram images. This resulted in drastic variable down-selection of the total size of the data matrix. Without this, it was not possible to fit the data matrix into the 32 GB of RAM in a standard personal computer. This process was done with fixed parameters for all echelle images of each sample. The purpose of this process was to mutually compare all image classes (*i.e*., sample — *e.g*., O100a, O101a, O101b, *etc*.) and to find pixel value differences between these image classes. This operation was performed by creating a median image for each image class (echellograms obtained from the measurement of each sample were organized in an array over one another, and the median was computed for the respective pixels from all echellograms). After that, the median image for each class was compared with other median images to obtain the difference image. In our case, where we have N = 50 image classes, we needed to perform 1225 comparisons to obtain 1225 difference images. Similar data analysis was performed when the sample set was sorted according to the ore type of each sample. The data set was then distributed into 13 classes, and multivariate projection and classification were applied to the data as in the previous case of 50 classes.

In the next step, a single maximal image containing the maximal pixel values of previous difference images was calculated. The maximal image contained areas with high pixel intensities, and in the middle of each of these areas, a point that denotes coordinates of a pixel is created. According to those selected points, attributes (*i.e*., important pixels) for machine learning are created. The images were processed, and their dimension was reduced from an initial 1024 × 1024 pixels to 225, 86 or even 21 most-important pixels. The selected attributes, which describe the pixels with changing intensity through image classes, are the output of the image pre-processing. Then, two data matrices (training and test) from these attributes were constructed with the ratio of 70:30 spectra per sample. Rows were assigned to individual measurements and columns to attributes/selected points in the echellogram. The data matrix contained data extracted from all image classes. These data sets were used for training and evaluation, which is described further.

### MVDA algorithms

Principal Component Analysis (PCA) is a linear MVDA algorithm of the least square sense^19^. Due to its simplicity, it is the most widespread algorithm, used broadly, not only in the spectroscopic community. In PCA computation, a multi-dimensional space is reduced into so-called principal components (PCs) that represent linear combinations of the original variables. Each PC is represented by scores (points in the new coordinate system substituting individual data/spectra) and loadings (giving weights/importance of individual variables). Moreover, each PC possesses a certain amount of the variance within the data set; the first PC possess the largest amount of the withdrawn variance from the original data. PCA was used for visualization of the obtained data set and investigation of the spectral features responsible for the clustering of the data.

We used two artificial intelligence machine learning algorithms for the classification of both data sets (1D spectra and truncated 2D echellograms). The first well-known supervised algorithm is called Support Vector Machine (SVM)^31^. This non-linear algorithm performs classification by creating N-dimensional hyperplanes, which separate data into the defined number of classes. SVM is usually used for the classification of more complex non-linear problems. All the parameters and configurations were determined experimentally, where all reasonable parameter values were tested and evaluated using the grid search method. The accuracy was evaluated as the number of true positive and negative classifications divided by the size of the data set. Because of the nature of the data, it was presumed that a linear kernel would provide the most reliable values, which was confirmed by the experiment. The linear kernel accuracy outperformed values achieved with radial, dot, polynomial (degrees 2 to 4), and ANOVA kernels (results not shown). For the complexity parameter, we preferred a value with the lowest possible complexity and reasonable accuracy. A lower complexity number usually leads to a model that is less prone to be over-fitted. It was also shown that in this particular case (SVM with the linear kernel), further optimization techniques (feature selection) had no positive effect on the resulting accuracy. The SVM algorithm was used with a linear kernel, and the “complexity” parameter was set to 5.

The second classification algorithm is Level 2 Regularized Logistic Regression (L2R LR)^32^. This linear algorithm was chosen for its simplicity and as a contrast to non-linear SVM, since it is generally accepted that non-linear algorithms yield higher classification figures of merit. The algorithm is analogous to linear regression (LR), but with the difference that LR uses classes for learning instead of numerical values. This type of LR uses a regularized function set to Level 2. In the L2R LR algorithm, the parameter “cost of constraints violation” was set to 5. We used both learning algorithms (SVM, LR) with the same parameters on 1D and 2D data. The data matrices were *a priori* column-wise normalized to range [−1,1]. These two learning algorithms work with given data matrices and their corresponding classes. The knowledge is trained, and then, the data not used for training are applied in this model to determine the final accuracy of the model. El Haddad *et al*.^20^ suggested using figures of merit for easier comparison of performances of the exploited LIBS systems and consecutive data processing algorithms.

In general, the MVDA model creation is extensive, being strongly dependent on the data matrix size (number of samples and variables). However, the classification of unknown data exploiting the already established model can yield a significantly higher repetition rate. The classification MVDA model can be established prior to the analysis and thus will not result in any significant delays during the classification or quantification itself.
